# Coronaviruses in diarrheic pigeons and the first determination of the complete genome of a new pigeon gammacoronavirus

**DOI:** 10.1038/s41598-025-03252-9

**Published:** 2025-05-31

**Authors:** Ewa Łukaszuk, Daria Dziewulska, Arvind Varsani, Tomasz Stenzel

**Affiliations:** 1 Department of Poultry Diseases, Faculty of Veterinary Medicine, University of Warmia and Mazury in Olsztyn, ul. Oczapowskiego 13, 10-719 Olsztyn, Poland; 2 Genomed S. A., Warsaw, Poland; 3 Biodesign Center for Fundamental and Applied Microbiomics, Center for Evolution and Medicine, School of Life Sciences, Arizona State University, Tempe, USA; 4 Structural Biology Research Unit, Department of Integrative Biomedical Sciences, University of Cape Town, Observatory, Cape Town, South Africa

**Keywords:** Coronavirus, ddPCR, Gammacoronavirus, Oxford nanopore sequencing, Pigeon, Phylogenetic analysis, Complete genome, Molecular biology, Viral infection, Phylogenetics, Virology

## Abstract

Members of *Orthocoronavirinae* subfamily are common among avian species and responsible for diseases in poultry. Unfortunately, knowledge of their diversity and potential pathogenic influence in pigeons is so far scarce. This study describes the first determination of a complete genomic sequence of a member of *Gammacoronavirus* genus in pigeons, likely representing new species within the *Igacovirus* subgenus. The newly obtained sequence appears to be closely related to partial gammacoronavirus sequences identified in pigeons from Poland, Spain, Finland and China, which highlights the wide geographical distribution of this virus. Direct quantitative analysis was performed in sampled pigeons to assess the potential connection of the presence of the virus and the occurrence of enteric disease, and while no statistically significant difference in the viral genome copy numbers was found between the diseased and healthy group, the similar detection rate in both groups is noteworthy. This result obtained from the analysis of 153 cloacal swab samples suggests that the presence of the virus likely is not associated with the occurrence of disease in their host. The study accentuates the need to further study the coronaviruses of avian origin especially considering the seemingly often occurrence of asymptomatic coronaviral infections and their broad geographical distribution.

## Introduction

### Coronavirus biology and epidemiology

Coronaviruses are members of the *Orthocoronavirinae* subfamily which currently consists of 4 genera, 26 subgenera and 57 species of enveloped, positive-sense ssRNA viruses with 26–32 kb linear genomes and 120–160 nm capsids decorated with characteristic surface projections resembling a solar corona in electron microscopy^1–3^. Coronaviruses are commonly found in multiple mammalian and avian species, with members of *Deltacoronavirus* and *Gammacoronavirus* genera infecting almost exclusively birds and often being associated with pathology of respiratory and gastrointestinal tract as well as with asymptomatic infections^3–5^. While infectious bronchitis virus (IBV; species *Gammacoronavirus galli*) occurring in chickens is undoubtedly the most known and thoroughly described avian-infecting representant of *Orthocoronavirinae*, coronaviruses are widely spread among many bird species, including poultry, wild birds and pigeons as well.

### Coronaviruses in pigeons

There have been multiple reports of pigeons as hosts of coronaviruses, although no association between the infection and the occurrence of clinical disease was established in most cases^6–15^. Enteric diseases are some of the most important health issues in racing pigeons, leading to impaired absorption and poor racing performance. Because coronaviruses are a prevalent finding in the gastrointestinal tract of multiple avian species including pigeons^5^, exploring their potential impact on the occurrence of enteric signs is of importance.

### Objectives of the study

This study is a part of a broader objective to identify viruses in the gastrointestinal tract of young racing pigeons of varying health status. We performed long read sequencing on cloacal swab samples to determine the viral biodiversity and identify viruses that might be involved in enteric diseases. Based on the identification of a coronavirus, we developed quantitative methods to help us determine any potential relationship between the presence of the coronavirus and the clinical symptoms. We determine the complete genome of the gammacoronavirus present in the intestine of symptomatic pigeon from Poland and explore how it might be connected to health status of the host. This is a continuation of our previous work, which involved screening of racing pigeons of different age and health status with pancoronavirus nested PCR, but did not include quantification of the virus^13^.

## Methods

### Ethics statement

The protocols for this study were reviewed and approved by the Local Ethics Committee on Animal Experimentation of University of Warmia and Mazury (UWM) in Olsztyn, Poland (resolution No 41/2019). All sampling was undertaken during a standard clinical examination of birds being patients of Department of Poultry Diseases, Faculty of Veterinary Medicine, UWM in Olsztyn, Poland. Permission was obtained from the owners to collect cloacal swab samples from the pigeons and to use them in the study. The fecal samples, used for qualification of birds for the study, were provided by the owners. The sampling was non-invasive and all pigeons were alive and unharmed after the sample collection. All methods were carried out in accordance with relevant guidelines and regulations and are reported in accordance with ARRIVE guidelines.

### Sample collection

The qualification of pigeons for sampling is described in detail by Łukaszuk et al.^16,17^. Cloacal swab samples were collected from 90 pigeons originating from 24 flocks in which signs of enteric disease were recently observed (study group) and from 63 healthy pigeons originating from 13 flocks without recent outbreaks of disease (control group). All pigeons tested were under 1 year of age and free of pathogenic bacteria, fungi and/or parasites based on fecal examination. After the sampling, the individual swabs were submerged in universal liquid transport medium for viruses as well as chlamydia, mycoplasma and ureaplasma (Copan Diagnostics, Murrieta, California, USA) and the medium was further divided into two parts and stored at − 80 °C until the analysis.

### Sequencing and bioinformatic analyses

#### Oxford nanopore sequencing

One half of the transport medium was enriched for viral particles by filtration with 0.8 µm polyethersulphone spin filters (Sartorius, Goettingen, Germany) and subjected to nuclease treatment and nucleic acid extraction. The DNA and RNA libraries were generated as previously described^18–20^. The libraries were run on Oxford nanopore GridION X5 sequencer with a R9.4.1 flow cell in combination with Rapid Barcoding Kit SQK-RBK110-96 used for library preparation (Oxford Nanopore Technologies Ltd., Oxford, United Kingdom) for 24 h. Oxford Nanopore Sequencing was performed in PathoSense laboratory (Oxford Nanopore Technologies Certified Service Provider, Merelbeke, Belgium).The resulting raw reads were transformed to bases with Guppy v7.1.4 and in-house bioinformatic pipelines were used to quality filter and taxonomically classify the sequences. As a final step, the viral genomic sequences, from each classified taxon separately, were de novo assembled with Canu v2.2^21^ and Medaka v1.4.1 tools (Oxford Nanopore Technologies Ltd., Oxford, United Kingdom). BLASTx^22^ was used to identify viral-like sequences against the Viral RefSeq protein database (release 210) downloaded from GenBank database (NCBI). The raw reads were mapped to the consensus virus genomes using Minimap2 v. 0.2-r123^23^. This mapping was subsequently examined and any discrepancies in the mapping and corrected as necessary.

#### Genome annotation and phylogenetic analyses

Open reading frames (ORFs) were determined in the coronavirus sequence with Find ORFs tool and annotated with Annotate by BLAST tool, both available in the Geneious Prime software v. 2025.0.3 (Dotmatics, Boston, Massachusetts, USA). The putative locations of conserved domains in replicase polyprotein were identified based on alignment with other avian coronavirus sequences (M95169, EU095850, MK359255 and KM454473). Using the BLAST search^22^ we identified similar sequences in the GenBank database (NCBI). In this manner, two gammacoronavirus sequence datasets were created – one composed of complete genomic sequences and the second of complete and partial sequences of varying length, including RNA-dependent RNA polymerase (RdRp) fragment. The sequences in both sets were aligned with MAFFT method^24^ and those in the second set were trimmed to the length of the shortest sequences and translated to amino acids, all in Geneious Prime software. The sets were then used to generate pairwise identity matrices with SDT v1.3 software^25^. Then, maximum likelihood phylogenetic trees using substitution models deemed most appropriate with Find DNA/protein models tool in MEGA 11 software^26^ were inferred with 1000 bootstrap replicates using IQ-TREE 1.6.12 software^27,28^. The phylogenetic trees were visualized with iTOL v6 software^29^. Finally, the newly obtained and annotated complete sequence of pigeon gammacoronavirus was deposited in GenBank (NCBI) under accession number PQ679936.

#### Recombination analysis

RDP5 software was used to identify any evidence of recombination with default parameters and the following methods: RDP, GENECONV, BOOTSCAN, MaxChi, Chimaera, SiScan, and 3SEQ^30–37^. Only recombination events detected by a minimum of three methods with *p* < 0.05 coupled with phylogenetic support were determined to be plausible.

### Quantitative analysis

#### RNA extraction and reverse transcription

RNA was extracted from the second part of the transport medium using Total RNA Mini Plus kit (A&A Biotechnology, Gdańsk, Poland) according to manufacturer’s instructions. The extracted RNA was eluted in 50 μl of nuclease-free water prior to determining the purity and concentration using a NanoDrop 2000 Spectrophotometer (Thermo Fisher Scientific, Waltham, Massachusetts, USA). Then, cDNA was synthesised by performing reverse transcription with 8 µl of RNA and 2 µl of 5X PrimeScript RT Master Mix (Takara Bio, Kusatsu, Japan). Finally, the samples were frozen at − 80 °C until analyzed further.

#### TaqMan quantitative PCR

A TaqMan qPCR assay was developed to test the samples for presence of pigeon gammacoronavirus. Primers (forward: 5′-ATGTAAAGCCTGGTGGGACT-3′ and reverse: 5′- AGACGCGCAACATTAGCTGA-3′) and probe (5′-[HEX]TGGTGATGCCACAACTGCTT [BHQ-1]-3′) were designed with Design New Primers tool available in Genious Prime software, using the sequence determined in this study as well as sequences acquired from GenBank: OM366031, OM366013, MK617492, KP033084 and KT222633. The assay targets a 100 bp fragment of RdRp gene. The reaction mixture was prepared by mixing 10 µl of TaqMan™ Fast Universal PCR Master Mix (Thermo Fisher Scientific, Waltham, Massachusetts), 1.8 µl of both 10 µM primers, 2 µl of 2.5 µM probe, 1.4 µl of nuclease-free water and 3 µl of cDNA. The conditions of the reaction carried out in the LightCycler® 96 System thermocycler (Roche, Basel, Switzerland) were as follows: 95 °C for 30 s, 40 cycles of 95 °C for 15 s and 59 °C for 40 s.

Specificity and sensitivity of the developed assay was assessed before testing the samples. To assess the specificity, the reaction was run on samples containing genetic material of other known pigeon viruses, such as pigeon rotavirus A (PP849436), pigeon astrovirus (PP478075), pigeon picornavirus B (PP735155) and megrivirus B (PP735150), as well as other avian coronaviruses, including infectious bronchitis virus (mass-like strains and variant strains) and gull deltacoronavirus. The coronavirus strains were kindly provided by Prof. Katarzyna Domańska-Blicharz (National Veterinary Institute, Puławy, Poland) and the other strains originated from the collection of Department of Poultry Diseases, Faculty of Veterinary Medicine, UWM in Olsztyn, Poland.

The sensitivity was determined based on a standard curve of a control we developed as follows. Using Geneious Prime software, we designed primers (forward: 5′-TGTCTTGCCCACCATAACTCAG-3′ and reverse: 5′-AACCCAGCACTTTGAGTCG-3′) targeting a 802 bp fragment that spans the qPCR assay region. The reaction mixture consisted of 10 μl of HotStar TaqPlus DNA Polymerase (Qiagen, Hilden, Germany), 0.1 μl of both 100 μM primers, 6.8 μl of nuclease-free water and 3 μl of pigeon coronavirus cDNA. The PCR reaction was carried out in a Mastercycler thermal cycler (Eppendorf, Hamburg, Germany) with the following thermal cycling conditions: 95 °C for 5 min, 40 cycles of 94 °C for 1 min, 56 °C for 1 min and 72 °C for 1 min, then 72 °C for 10 min. The resulting amplicons were purified using the Clean-Up kit (A&A Biotechnology, Poland) and NanoDrop 2000 Spectrophotometer (Thermo Fisher Scientific, Waltham, Massachusetts, USA) was used to determine the concentration and purity. The gene copy number was calculated with a copy number calculator (Genomics and Sequencing Center, University of Rhode Island, Kingston, Rhode Island). Finally, TaqMan qPCR was performed with series of decimal dilutions from 3.7 × 10^7^ to 3.7 × 10^3^ copies of the amplicon/μl used as a template, and all samples were tested in triplicate.

The proper testing of the samples was then conducted, using a sample from the collection of Department of Poultry Diseases, Faculty of Veterinary Medicine, UWM in Olsztyn, Poland as a positive control (OM366031). All samples were tested in duplicate and those with Cq of 35 or less were considered positive.

#### Droplet digital PCR

To directly quantify the loads of pigeon gammacoronavirus in samples positive by TaqMan qPCR, we developed a droplet digital PCR (ddPCR) method, using the same primers, probe and positive control as for qPCR. First, 22 μl of reaction mixture was prepared by mixing 11 μl of ddPCR Supermix for Probes, 1.98 μl of both primers and 2.2 μl of probe, 1.84 μl of RNase-free water and 3 μl of cDNA. Next, droplet emulsions were prepared in the same way as in our previous studies^16,17^. Then, the reaction was performed in a C1000 Touch Thermal Cycler (Bio-Rad laboratories) and the conditions were as follows: 95 °C for 10 min, 40 cycles of 94 °C for 30 s and 50 °C for 1 min, and 4 °C for 30 min; every step had a ramp rate of 2 °C/s. QX 200 Droplet Reader was then used to calculate the number of coronaviral amplicons in the droplets. All reagents and devices used for ddPCR were manufactured by Bio-Rad (Hercules, California, USA). Resulting counts were presented as mean number of viral genome copies ± standard deviation per 20 µl of the sample, and in the case of samples diluted prior to ddPCR, the values were multiplied by the dilution value.

#### Statistical analysis

Based on expected values, V-square test (V^2^) was deemed the most appropriate to assess the correlation between the detection rate of the virus and the health status of the pigeons. As for the correlation of the amount of viral genetic material in the positive samples with the health status of the pigeons, it was tested using Mann–Whitney U non-parametric test. Differences were considered significant with *P* < 0.05 and the analyses were done in Statistica 13 software (Statsoft, Cracow, Poland).

## Results and discussion

### Bioinformatic analysis

#### Genome organization

**Fig. 1:**
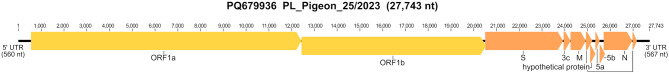
Genome organization of pigeon gammacoronavirus determined in this study. Explanation of abbreviations: UTR – untranslated region, ORF – open reading frame, S – spike protein, 3c—3c protein, M – membrane protein, 5a – 5a protein, 5b – 5b protein, N – nucleocapsid protein.

Through a long read metagenomic approach on swab samples acquired from young racing pigeons, we identified several viruses which could be involved in an occurrence of enteropathy^16,17,38–40^. In this report, we describe the identification and determination of the complete genome sequence of a virus belonging to the *Coronaviridae* family. This sequence was identified in a single pigeon from the study group exhibiting signs of enteric disease. This pigeon coronavirus is 27,743 nt in length and has genome organization typical of members of *Gammacoronavirus* genus, consisting of multiple open-reading frames (ORFs) and the following regions can be distinguished: replicase gene organized in ORF1a and ORF1b, spike gene, 3c protein gene, membrane gene, 5a protein gene, 5b protein gene, nucleocapsid gene and a few genes encoding hypothetical proteins. Untranslated regions (UTRs) of 560 nt and 567 nt in length can be recognized on 5′ and 3′ ends of the sequence, respectively. The schematic illustration of the genome is provided in Fig. 1 and a summary of the genes is provided in Table 1.

**Table 1:** Gene characterization of complete genomic sequence of pigeon gammacoronavirus obtained in this study.

Gene	Length [nt]	Start codon	Stop codon	GC content [%]
Replicase protein	11,880 + 8103	AUG	UAG	36.7
Spike protein	3420	UUG	UGA	36.1
3c protein	303	AUG	UAA	34.3
Membrane protein	678	AUG	UAA	39.4
Hypothetical protein 1	285	AUG	UAG	34.0
Hypothetical protein 2	234	AUG	UGA	38.0
5a protein	192	AUG	UGA	32.8
5b protein	240	AUG	UAA	44.6
Nucleocapsid protein	1254	AUG	UAA	46.3
Hypothetical protein 3	192	AUG	UAA	29.7

#### Phylogenetic analysis

**Fig. 2:**
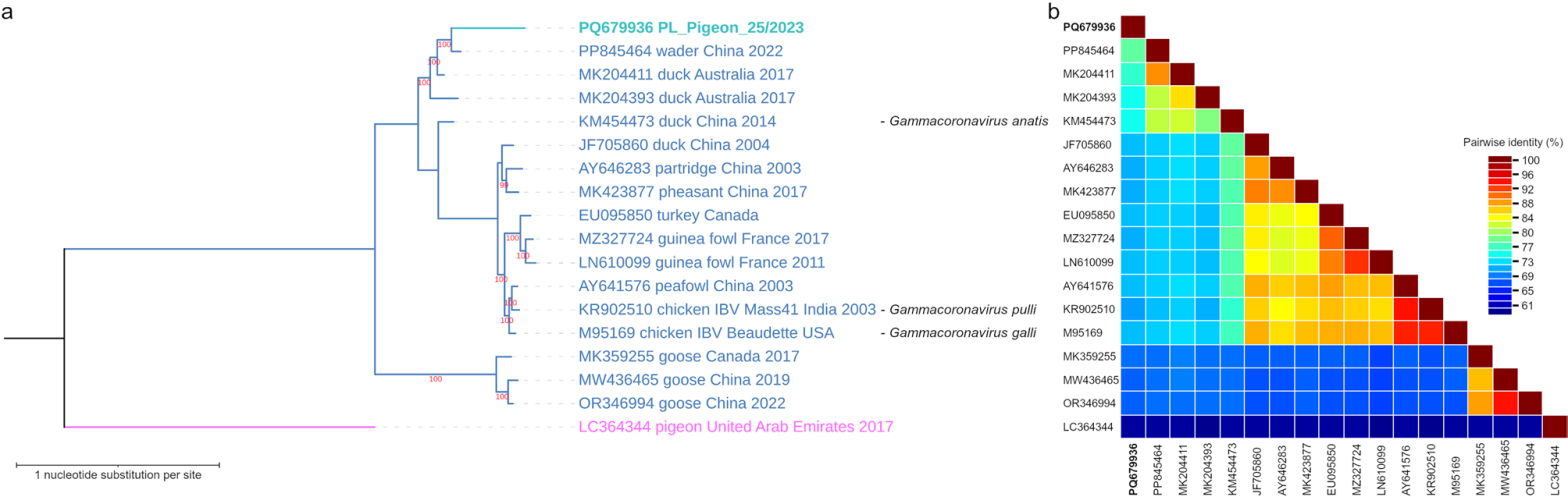
(**a**) Maximum likelihood phylogenetic tree using the GTR + G substitution model, inferred of complete genomic sequences of members of *Gammacoronavirus* genus either obtained in this study or acquired from GenBank database. All sequences are labeled with accession number, host, country of origin and year of sampling, if available. IBV sequences are additionally labeled with strain name. The label of the sequence obtained from a pigeon within the framework of this study is written in bold and in turquoise color, while the labels of the sequences originating from other avian species are written in blue color. The tree has been rooted with the sequence of a member of *Deltacoronavirus* genus acquired from a pigeon and its label is written in pink. (**b**) Pairwise identity matrix composed of the same complete genomic sequences as in the phylogenetic tree.

BLAST search revealed that the pigeon coronavirus (PQ679936) from this study indeed likely belongs to the *Gammacoronavirus* genus, with the most similar sequence being a virus unassigned to any subgenus and species, identified from a great knot (*Calidris tenuirostris*) from China (accession number PP845464)^41^, sharing 77.9% nucleotide identity with E-value of 0 and 96% genome coverage. The genetic relationship based on the phylogeny of the complete genome is provided in Fig. 2. In the phylogenetic tree based on the complete genomes, the pigeon coronavirus clusters with other gammacoronaviruses and, more specifically, branches with great knot derived gammacoronavirus (PP845464) and with two sequences identified in ducks from Australia (MK204411 and MK204393) that are certain members of the *Igacovirus* subgenus but are unclassified at a species level. The *Igacovirus* subgenus currently consists of only three species, *Gammacoronavirus anatis*, *Gammacoronavirus galli* and *Gammacoronavirus pulli,* whose members are duck coronavirus DK/GD/27/2014 (KM454473), infectious bronchitis virus (M95169) and infectious bronchitis virus Ind-TN92-03 (KR902510), respectively (Fig. 2a). Infectious bronchitis virus (M95169) and infectious bronchitis virus Ind-TN92-03 (KR902510) share 93.6% genome-wide pairwise identity and these two share 76.1–77.0% identity with duck coronavirus DK/GD/27/2014 (KM454473). These three that each represent a species share 70.4–74.7% pairwise identity with the pigeon coronavirus (PQ679936) from this study (Fig. 2b).

Comparison of the complete genomes might not be the most adequate considering possible huge differences across the divergent regions of the genome, hence comparison of a conserved region such as RdRp gene may be more appropriate for a more robust classification. The RdRp sequence most similar to that of the pigeon coronavirus from this study (PQ679936) is the one from the great knot (PP845464), sharing 94.4% amino acid identity. The representative sequences of classified species within *Igacovirus* subgenus, namely *Gammacoronavirus anatis* (KM454473), *Gammacoronavirus galli* (M95169) and *Gammacoronavirus pulli* (KR902510) shared 93.5%, 87.8% and 82.4% RdRp amino acid identity, respectively, with that of the pigeon coronavirus from this study (PQ679936). The analysis of other conserved domains in the replicase polyprotein is summarized in Table 2. Taking into account the species demarcation threshold of ≤ 90% amino acid identity in the conserved replicase domains described by ICTV *Coronaviridae* study group^42^, the pigeon coronavirus sequence determined in this study may represent a diverse member of the species *Gammacoronavirus anatis*. Nonetheless, the earlier described genome analysis suggests that the pigeon derived coronaviruses may form a distinct species, as is the case for Infectious bronchitis virus (M95169) and infectious bronchitis virus Ind-TN92-03 (KR902510) that share 92.9% pairwise identity in the RdRp amino acid sequences and 93.6% nucleotide pairwise identity at a genome level and are considered members of separate species.

**Table 2:** Pairwise identities of amino acid sequences of conserved domains in replicase polyprotein, shared by pigeon gammacoronavirus determined in this study and the representants of the three species in *Gammacoronavirus* genus.

		*Gammacoronavirus galli* (M95169) (%)	*Gammacoronavirus pulli* (KR902510) (%)	*Gammacoronavirus anatis* (KM454473) (%)
nsp3 (ADRP)	Pigeon gammacoronavirus (PQ679936)	55.7	54.1	57.6
nsp5 (3CL^pro^)		69.7	68.7	70.0
nsp12 (RdRp)		87.6	82.4	93.4
nsp13 (Hel)		90.8	83.7	94.8
nsp14 (ExoN)		87.5	83.3	90.3
nsp15 (NendoU)		81.1	79.9	84.0
nsp16 (O-MT)		78.8	70.9	82.7

**Fig. 3:**
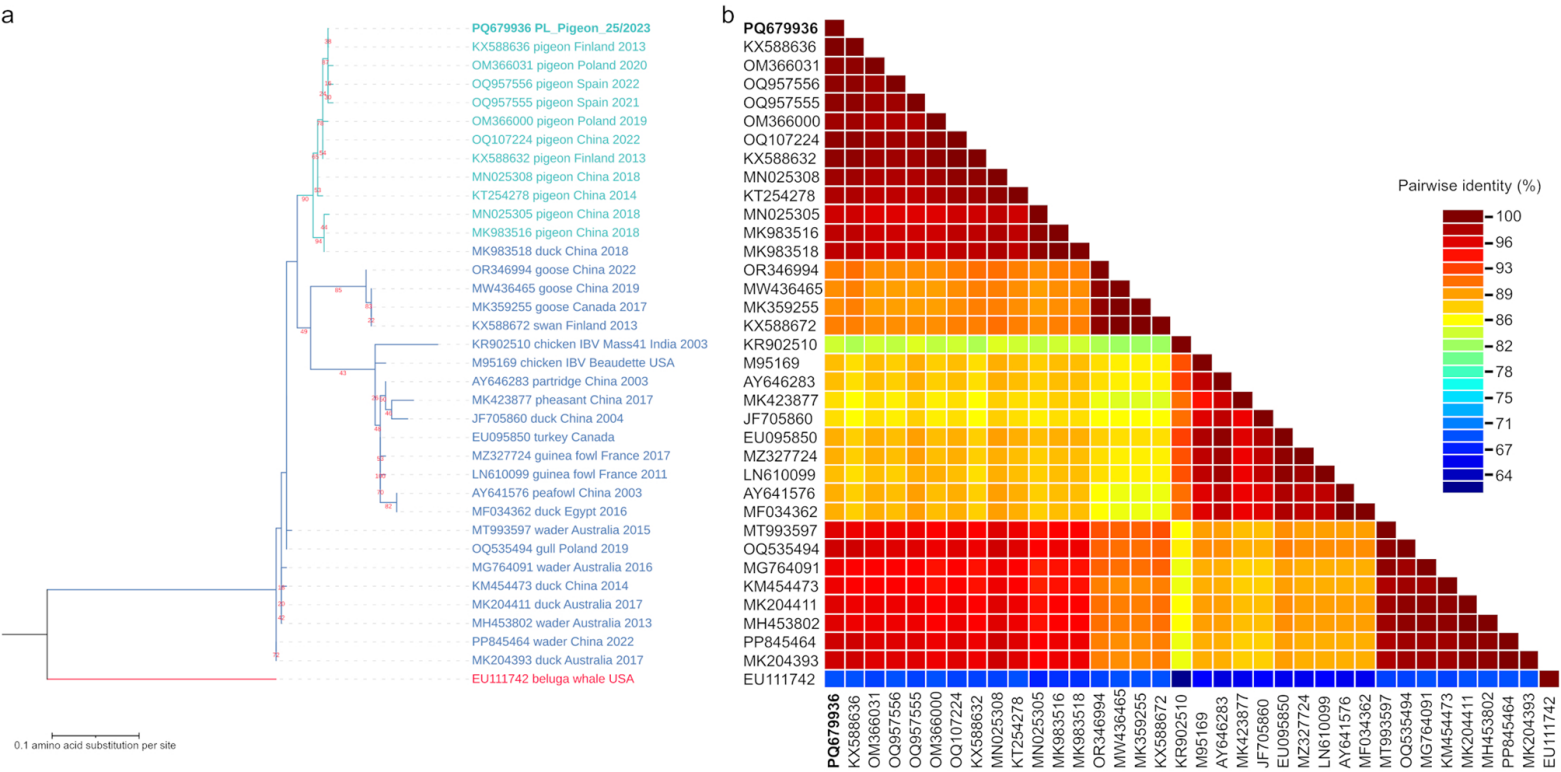
(**a**) Maximum likelihood phylogenetic tree using the LG + G substitution model, inferred of RNA-dependent RNA polymerase sequences of 167 amino-acids in length. The sequences used are those of members of *Gammacoronavirus* genus either obtained in this study or acquired from GenBank database. All sequences are labeled with accession number, host, country of origin and year of sampling, if available. IBV sequences are additionally labeled with strain name. The labels of the sequences originating from pigeons are written in turquoise color, while the labels of the sequences originating from other avian species are written in blue color. The label of the sequence obtained within the framework of this study is additionally written in bold. The tree has been rooted with gammacoronavirus sequence acquired from a beluga whale and its label is written in red. (**b**) Pairwise identity matrix composed of the same amino-acid sequences as in the phylogenetic tree.

Although the full genome of pigeon gammacoronavirus had not been determined before, multiple additional sequences consisting of partial RdRp gene of unclassified members of *Gammacoronavirus* genus are available in GenBank and we include these in the analyses. The phylogenetic tree composed of amino acid sequences spanning a 167 aa RdRp region is provided in Fig. 3a. It can be noted that the pigeon gammacoronavirus sequence from our study forms a separate cluster with other pigeon gammacoronavirus sequences originating from pigeons from Poland, Spain, Finland and China as well as pigeon-dominant gammacoronavirus sequence obtained from a duck from China. The 167 aa RdRp fragment of the sequence obtained in this study shares an average 98.8% amino acid identity with the sequences forming the pigeon cluster and 91.3% amino acid identity with the sequences used for this analysis that were obtained from non-pigeon species. The fragment of RdRp sequence most similar to that obtained in this study belongs to pigeon gammacoronavirus from Finland (KX588636) with 100% amino acid identity, while the sequence of great knot coronavirus from China (PP845464) had 97.0% amino acid identity within the analyzed RdRp fragment with the newly obtained sequence, which is the highest score among the non-pigeon-dominant strains. In turn, the least similar sequence is that of IBV Ind-TN92-03 (classical strain—Mass41) from India (KR902510) with 83.8% amino acid identity with the new gammacoronavirus sequence. All pairwise identities of the 167 aa RdRp fragments are provided as color matrix in Fig. 3b. The RdRp fragment amino acid phylogenetic tree confirms the close genetic relationship of gammacoronaviruses obtained from pigeons, regardless of the broad geographical spread of the places of origin.

In this study we determine the first complete gammacoronavirus genome originating from domestic pigeon. Partial sequences of pigeon-dominant gammacoronaviruses of *Igacovirus* subgenus have been identified in various countries, although these sequences covered no more than single genes^9,11,12,14,15^. The only other available complete genome of a representative of *Coronaviridae* family originating from a pigeon is that of a deltacoronavirus, detected in United Arab Emirates in 2017 (accession number LC364344)^10^.

#### Recombination analysis

We did not find any credible evidence of recombination in the sequences we analyzed. Recombination is considered an important mechanism of coronavirus evolution, which can lead to formation of new strains of varying pathogenicity, cell and tissue tropism and leading to broader host range^43,44^. In our case, a limited number and short length of pigeon-derived coronavirus sequences in public databases available might hinder the analysis.

### Quantitative analysis

All the viruses except for pigeon gammacoronavirus tested negative in the TaqMan qPCR, proving the specificity of the newly developed assay. The sensitivity of the assay was established as 3.7 × 10^3^ copies of the amplicon/μl and the characteristics of the standard curve were as follows: slope − 3.4773, efficiency 94%, error 0.27, Y-intercept 45.12 and R^2^ 1.00.

**Fig. 4:**
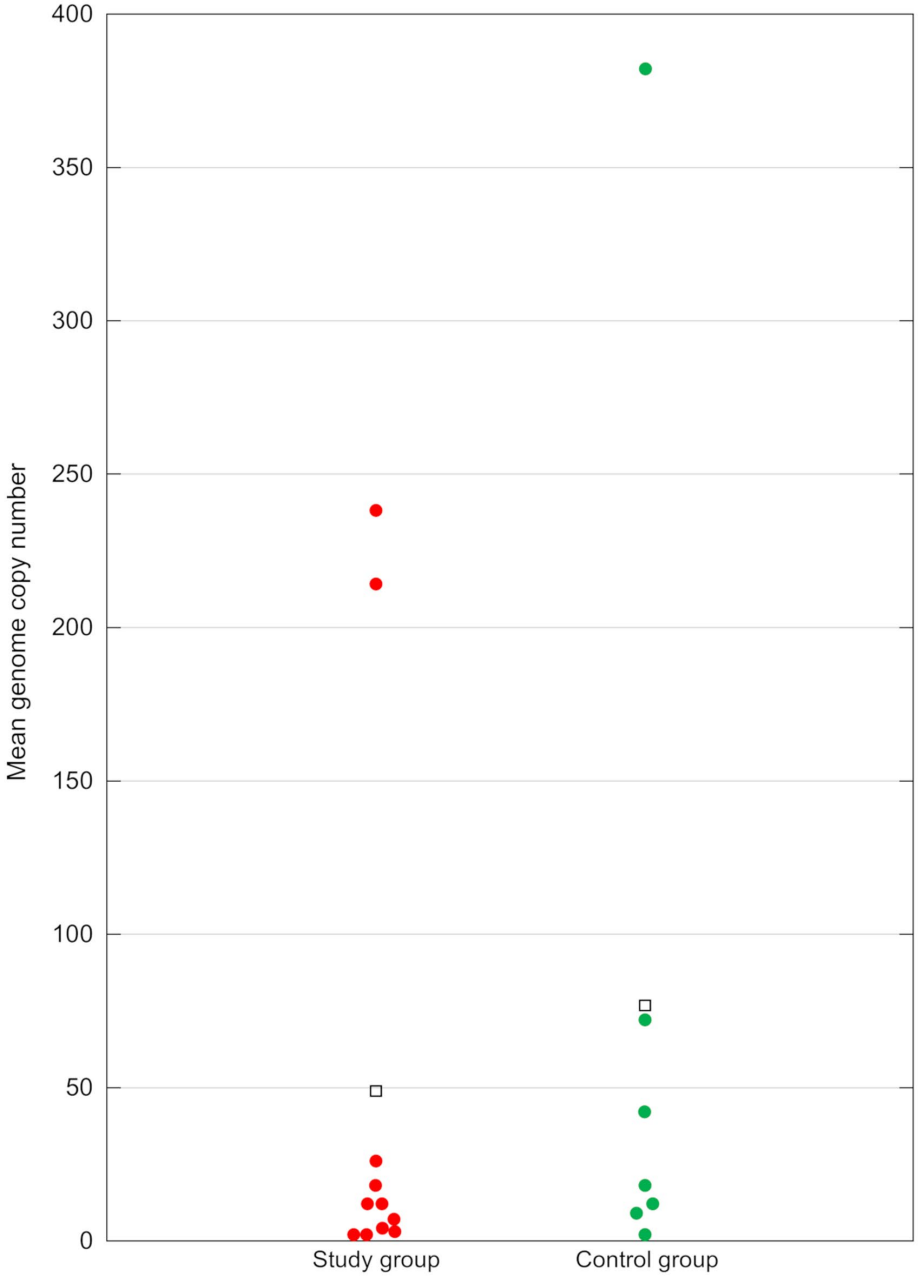
Graphical presentation of results of droplet digital PCR for pigeon gammacoronavirus. The genome copy numbers are calculated per 20 μL of each sample. Hollow squares represent the mean number of viral genome copies in each group tested.

Out of 90 samples in the study group, 11 (12.2%) and 7 samples out of 63 in the control group (11.1%) turned out to be positive for the pigeon gammacoronavirus using the TaqMan qPCR assay. The positive samples originated from 9 flocks from the study group and from 4 flocks from the control group. The statistical analysis revealed that the difference in the detection rate of the virus between the groups was insignificant (V^2^ = 0.04, P = 0.8343). The viral shedding detected with ddPCR was similar in both groups, measuring on average 48.91 ± 88.03 genome copies per 20 μl of the sample in the study group and 76.71 ± 136.77 genome copies per 20 μl of the sample in the control group (Fig. 4). This difference was also found insignificant with *P* = 0.3896. The results of the quantitative analysis indicate that the presence of pigeon gammacoronavirus in the examined birds probably had no correlation with the occurrence of enteric disease. This is consistent with the results of our preliminary research, where pigeon gammacoronavirus genome fragment was detected in pigeons with nested PCR and the difference in the rate of detection between the two groups of different health status was not large enough to be considered significant^13^. However, the relatively low number of samples qualified for the present study might limit the analysis. Unfortunately, it is not possible to unequivocally confirm or exclude the pathogenic effect of pigeon gammacoronavirus because efforts to culture it in vitro so far have been unsuccessful^6,11^. Lack of research regarding coronavirus infection in pigeons with signs of enteric disease hinders potential deliberations on this subject. While in recent years a few studies on surveillance of coronaviruses in pigeons have been carried out, none offers information on health status of the sampled pigeons^9–12,14,15^. Coronavirus infections in birds are often subclinical and such situation can also be observed in the case of pigeons tested in this study – genetic material of pigeon gammacoronavirus has been detected in 11.1% of asymptomatic birds. The overall detection rate of 11.8% is not very surprising, as prevalence in the recent studies ranges from 3.6% noted in pigeons in Finland, through 13.3% in Spain and 23.14% in China, to 26.5% in Poland^9,11,13,15^. Taking all the aforementioned research into account, it can be presumed that coronaviruses are widespread in the pigeon population across the world, although the lack of characteristic clinical presentation makes their detection rather incidental.

## Conclusions

In this study we were able to determine the first complete genomic sequence of pigeon coronavirus belonging to *Gammacoronavirus* genus, which is a substantial finding expanding the knowledge on avian-infecting coronaviruses. This sequence likely represents a distant member of an existing species or a member of a new species within the *Igacovirus* subgenus which will be determined by the ICTV *Coronaviridae* study group. Although quantitative analysis performed within the framework of this research did not provide any definitive conclusions regarding the potential pathogenicity of pigeon gammacoronavirus, its detection rate similar in diseased and apparently healthy individuals suggests asymptomatic infections with this virus may be common. The widespread and diversity of coronaviruses in different avian species is remarkable and it is thought that it might be a result of behavior such as roosting and migration, as well as certain characteristics of the immune system that facilitate viral spread^45^. New coronaviruses of avian origin are still being discovered and good understanding of their diversity and epidemiology is necessary to be able to assess what risks, if any, they may pose to domestic animals as well as humans. It is especially important considering the seemingly often occurrence of asymptomatic coronaviral infections. In case of gammacoronavirus detected in pigeons in Poland, the results of statistical analysis performed in this study suggest that the virus likely had no connection with the occurrence of disease in the hosts.

## Data Availability

The nucleotide sequence data obtained in this study were deposited in GenBank database under the accession number PQ679936 and can be found at the link https://www.ncbi.nlm.nih.gov/nuccore/PQ679936. The datasets generated and analyzed during the current study are available from the corresponding author on reasonable request.
