# Author Correction: Coronaviruses in diarrheic pigeons and the first determination of the complete genome of a new pigeon gammacoronavirus

**DOI:** 10.1038/s41598-025-11484-y

**Published:** 2025-08-19

**Authors:** Ewa Łukaszuk, Daria Dziewulska, Arvind Varsani, Tomasz Stenzel

**Affiliations:** 1 Department of Poultry Diseases, Faculty of Veterinary Medicine, University of Warmia and Mazury in Olsztyn, ul. Oczapowskiego 13, 10-719 Olsztyn, Poland; 2 Genomed S. A., Warsaw, Poland; 3 Biodesign Center for Fundamental and Applied Microbiomics, Center for Evolution and Medicine, School of Life Sciences, Arizona State University, Tempe, USA; 4 Structural Biology Research Unit, Department of Integrative Biomedical Sciences, University of Cape Town, Observatory, Cape Town, South Africa

Correction to: *Scientific Reports* 10.1038/s41598-025-03252-9, published online 31 May 2025

The original version of this Article contained an error in the order of the Figures. Figure 3 was published as figure 2, figure 4 was published as figure 3, and figure 2 was published as figure 4. As a result, the Figure legends were incorrect.

The original Figures 2, 3 and 4 and accompanying legends appear below.

The original Article has been corrected.

**Fig. 2:**
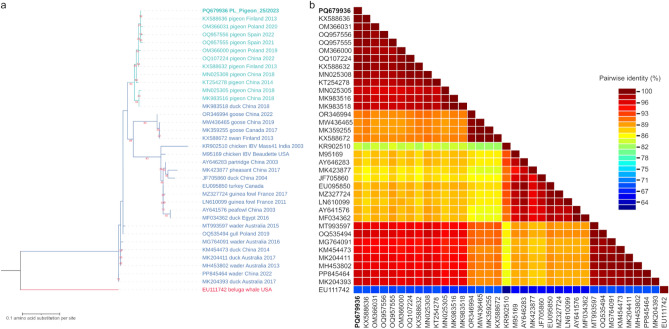
(**a**) Maximum likelihood phylogenetic tree using the GTR + G substitution model, inferred of complete genomic sequences of members of *Gammacoronavirus* genus either obtained in this study or acquired from GenBank database. All sequences are labeled with accession number, host, country of origin and year of sampling, if available. IBV sequences are additionally labeled with strain name. The label of the sequence obtained from a pigeon within the framework of this study is written in bold and in turquoise color, while the labels of the sequences originating from other avian species are written in blue color. The tree has been rooted with the sequence of a member of *Deltacoronavirus* genus acquired from a pigeon and its label is written in pink. (**b**) Pairwise identity matrix composed of the same complete genomic sequences as in the phylogenetic tree.

**Fig. 3:**
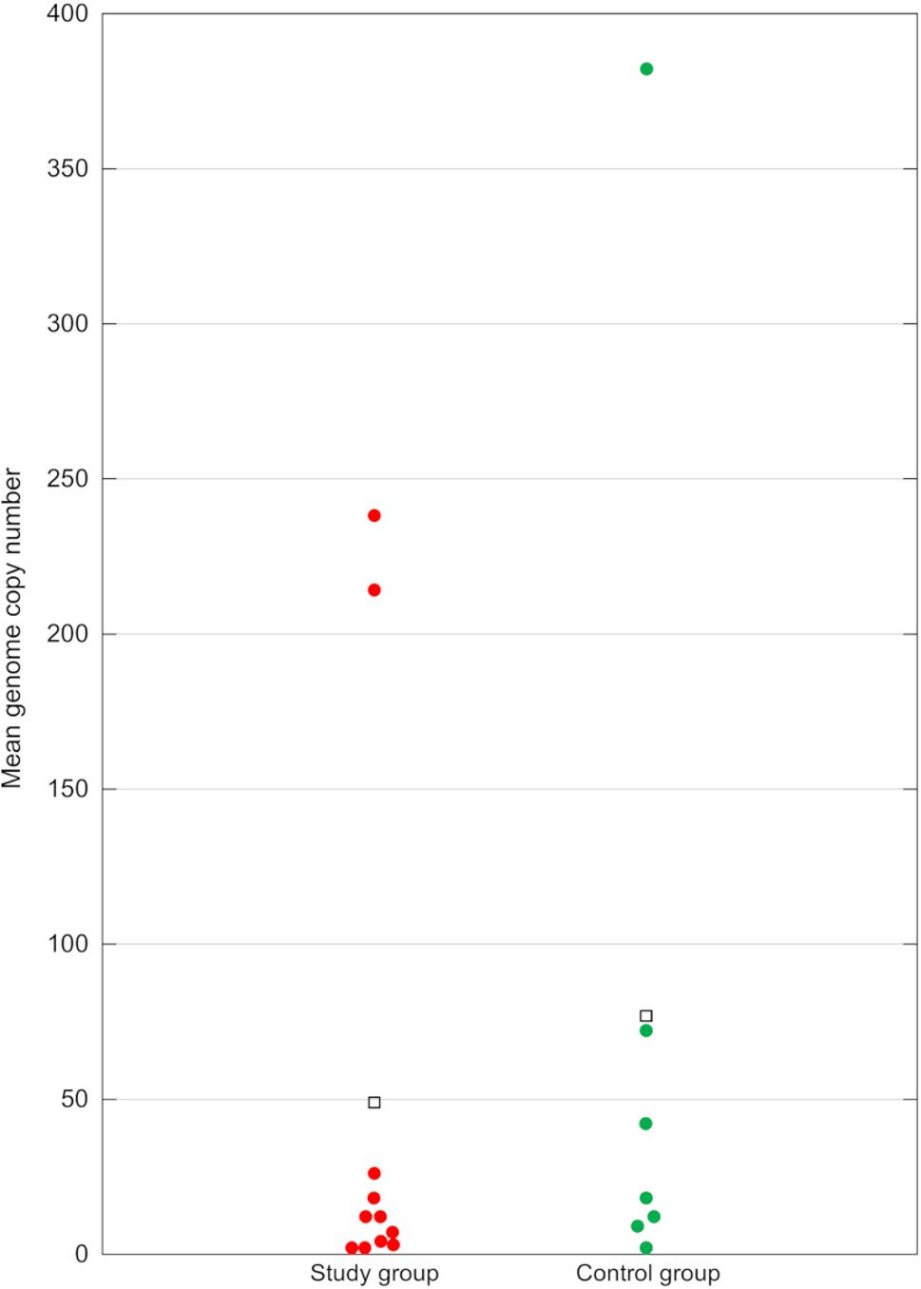
(**a**) Maximum likelihood phylogenetic tree using the LG + G substitution model, inferred of RNA-dependent RNA polymerase sequences of 167 amino-acids in length. The sequences used are those of members of *Gammacoronavirus* genus either obtained in this study or acquired from GenBank database. All sequences are labeled with accession number, host, country of origin and year of sampling, if available. IBV sequences are additionally labeled with strain name. The labels of the sequences originating from pigeons are written in turquoise color, while the labels of the sequences originating from other avian species are written in blue color. The label of the sequence obtained within the framework of this study is additionally written in bold. The tree has been rooted with gammacoronavirus sequence acquired from a beluga whale and its label is written in red. (**b**) Pairwise identity matrix composed of the same amino-acid sequences as in the phylogenetic tree.

**Fig. 4:**
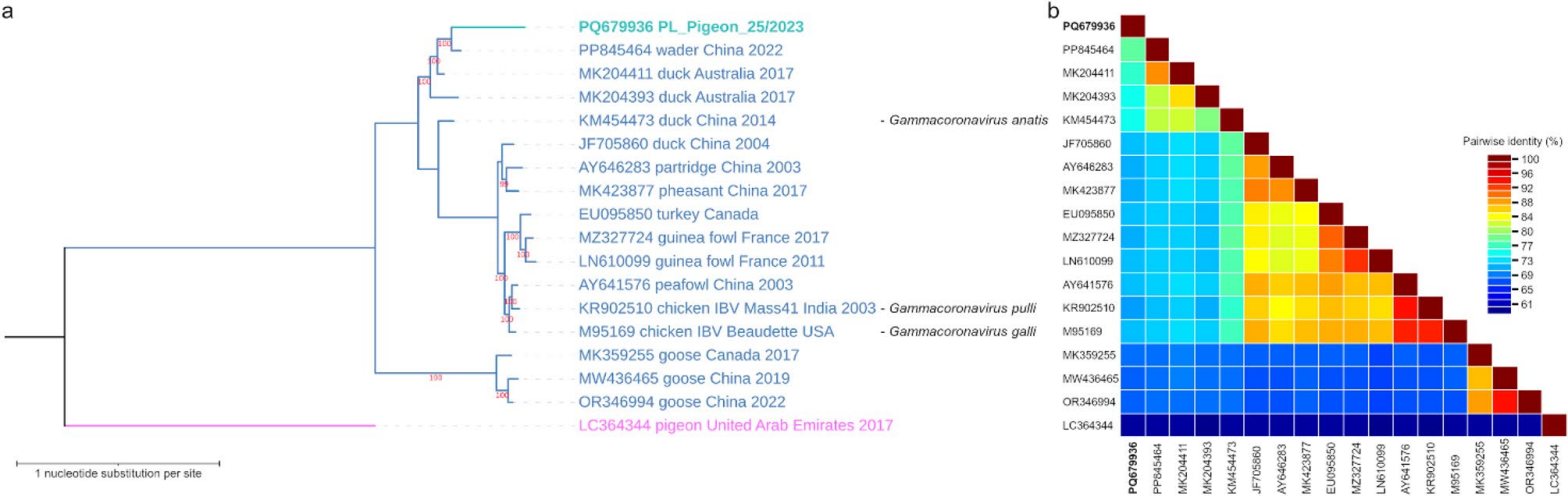
Graphical presentation of results of droplet digital PCR for pigeon gammacoronavirus. The genome copy numbers are calculated per 20 μL of each sample. Hollow squares represent the mean number of viral genome copies in each group tested.

